# Demand for Water-Soluble Vitamins in a Group of Patients with CKD versus Interventions and Supplementation—A Systematic Review

**DOI:** 10.3390/nu15040860

**Published:** 2023-02-08

**Authors:** Karolina Kędzierska-Kapuza, Urszula Szczuko, Hanna Stolińska, Dimitra Rafailia Bakaloudi, Waldemar Wierzba, Małgorzata Szczuko

**Affiliations:** 1 State Medical Institute of the Ministry of Interior and Administration in Warsaw, 137 Wołoska St., 02-507 Warsaw, Poland; 2 Center of Postgraduate Medical Education in Warsaw, Department of Gastroenterological Surgery and Transplantology, 137 Wołoska St., 02-507 Warsaw, Poland; 3 Department of Human Nutrition and Metabolomic, Pomeranian Medical University in Szczecin, 71-460 Szczecin, Poland; 4 Love Yourself Hanna Stolińska, 112 Sobieskiego St., 00-764 Warsaw, Poland; 5 Department of Medical Oncology, General Hospital of Thessaloniki “G. Papageorgiou”, Aristotle University of Thessaloniki, 54623 Thessaloniki, Greece; 6 Division of Oncology, Department of Medicine, University of Washington, Seattle, WA 98109-1023, USA

**Keywords:** chronic kidney disease, vitamins B, vitamin C, vitamin supplementation

## Abstract

Background: Increasingly, chronic kidney disease (CKD) is becoming an inevitable consequence of obesity, metabolic syndrome, and diabetes. As the disease progresses, and through dialysis, the need for and loss of water-soluble vitamins both increase. This review article looks at the benefits and possible risks of supplementing these vitamins with the treatment of CKD. Methods: Data in the PubMed and Embase databases were analyzed. The keywords “chronic kidney disease”, in various combinations, are associated with thiamin, riboflavin, pyridoxine, pantothenic acid, folates, niacin, cobalamin, and vitamin C. This review focuses on the possible use of water-soluble vitamin supplementation to improve pharmacological responses and the overall clinical condition of patients. Results: The mechanism of supportive supplementation is based on reducing oxidative stress, covering the increased demand and losses resulting from the treatment method. In the initial period of failure (G2-G3a), it does not require intervention, but later, especially in the case of inadequate nutrition, the inclusion of supplementation with folate and cobalamin may bring benefits. Such supplementation seems to be a necessity in patients with stage G4 or G5 (uremia). Conversely, the inclusion of additional B6 supplementation to reduce CV risk may be considered. At stage 3b and beyond (stages 4–5), the inclusion of niacin at a dose of 400–1000 mg, depending on the patient’s tolerance, is required to lower the phosphate level. The inclusion of supplementation with thiamine and other water-soluble vitamins, especially in peritoneal dialysis and hemodialysis patients, is necessary for reducing dialysis losses. Allowing hemodialysis patients to take low doses of oral vitamin C effectively reduces erythropoietin dose requirements and improves anemia in functional iron-deficient patients. However, it should be considered that doses of B vitamins that are several times higher than the recommended dietary allowance of consumption may exacerbate left ventricular diastolic dysfunction in CKD patients. Conclusions: Taking into account the research conducted so far, it seems that the use of vitamin supplementation in CKD patients may have a positive impact on the treatment process and maintaining a disease-free condition.

## 1. Introduction—CKD Causes and Metabolic Effects

Chronic kidney disease (CKD) often results from diabetes or hypertension, and less often, from glomerulonephritis, polycystic kidney disease, kidney stones, or chronic pyelonephritis. CKD is a major task for public health due to its high incidence and often subsequent progression to end-stage renal disease (ESRD), a higher risk of cardiovascular disease (CVD), and cardiovascular events, which represent the leading causes of death [1]. Explaining such a high risk of cardiovascular events is difficult because the cause is multifactorial.

### 1.1. Epidemiology and Classification

Modifiable risk factors include the following:

- Hyperglycemia;
- Hypertension;
- Hyperlipidemia;
- Hyperphosphatemia;
- Diets rich in animal protein.

Therefore, therapy based on dieting should focus on the following:

- Inhibiting kidney damage;
- Preventing malnutrition;
- Reducing the severity of metabolic disorders.

According to the KDIGO statement, there are five stages of CKD depending on GFR [2]:

- Stage 1—Normal or high GFR (GFR > 90 mL/min/1.73 m^2^);
- Stage 2—Mild CKD (GFR = 60–89 mL/min/1.73 m^2^);
- Stage 3A—Moderate CKD (GFR = 45–59 mL/min/1.73 m^2^);
- Stage 3B—Moderate CKD (GFR = 30–44 mL/min/1.73 m^2^);
- Stage 4—Severe CKD (GFR = 15–29 mL/min/1.73 m^2^);
- Stage 5—End-stage CKD (GFR < 15 mL/min/1.73 m^2^).

### 1.2. Metabolic Effects in CKD

The slow, irreversible, and progressive deterioration of kidney function, characterized by a reduction in the glomerular filtration rate, can have many complications. CKD increases the risk of heart and vascular diseases in patients, and CKD complications include anemia and bone diseases.

In the treatment of metabolic acidosis, the consumption of alkalis (fruit and vegetables) should increase in patients with CKD. In stages 3 and 4 of CKD, the bicarbonate level should be between 22 and 24 mmol/L (stable level). In the later stages of CKD, metabolic acidosis should be corrected with sodium bicarbonate (NaHCO3) supplementation. At stages 4/5, it is recommended that sodium bicarbonate tablets are administered 2–3 times a day. The dose is adjusted by controlling the alkali concentrations in the capillary blood. The alkali concentration should be maintained at >22 mmol/L [3].

### 1.3. Nutritional Status in CKD

Good nutritional status is roughly described as having a BMI of 23–26. Protein and energy malnutrition is present and anorexia occurs, depending on the source, in up to 50% of CKD stage 5 patients [4].

The definition of malnutrition according to the WHO (2020) refers to deficiencies, excesses, or imbalances in the intake of energy and/or nutrients. The term malnutrition covers two broad groups of conditions. The first is “malnutrition”—which includes stunted growth (short height for age), cachexia (low weight for height), underweight (low weight for age), and micronutrient and macronutrient deficiencies. The second is overweight, obesity, and diet-related diseases (such as heart disease, stroke, diabetes, and cancer) [5].

Cachexia is a complicated metabolic syndrome related to underlying illness and characterized by muscle mass loss with or without fat mass loss that is often associated with anorexia, an inflammatory process, insulin resistance, and increased protein turnover [6]. There is currently no effective pharmacological intervention to prevent or attenuate muscle wasting in catabolic conditions such as CKD.

In dialysis patients, symptoms of depression are associated with increased levels of IL-6. Depression can also lead to fatigue and appetite loss, which contribute to anorexia, weakness, physical inactivity, and overall protein energy wasting (PEW), leading to the initiation of a vicious cycle mechanism [7,8]. Protein energy wasting (PEW)—the concept of PEW—was proposed in 2007 by the International Society of Renal Nutrition and Metabolism (ISRNM) as a state of nutritional and metabolic derangements in patients with chronic kidney disease (CKD) characterized by simultaneous loss of systematic body protein and energy stores, ultimately leading to a loss of muscle and fat mass and cachexia [9]. More recent studies have focused on protein energy wasting (PEW), which is an implicit cause of sarcopenia and frailty. PEW is an energy-wasting condition that occurs in dialysis patients, and the principal causes of PEW are decreased eating and increased catabolism. It has lately been reported that decreased protein intake could be a risk factor for increased mortality in end-stage kidney disease patients. The incidence of PEW was reported to reach up to 14% in this group of patients [10]. Dialysis patients are a unique group in which the phenomenon of the inverted survival curve in relation to BMI has been observed. Patients with a higher BMI survive longer than those with a low BMI. The occurrence of “malnutrition–inflammation complex syndrome” (MICS) in dialysis patients may also justify the possibility of reverse epidemiology in them [11]

Recently, an update to the KDOQI Clinical Practice Guideline for Nutrition in CKD was developed in 2020. The guideline not only involves end-stage kidney disease or CKD stage 1–5 patients, but also kidney graft recipients [12].

### 1.4. Peritoneal Dialysis and Hemodialysis vs. Macronutrients

During hemodialysis sessions, there is not only a problem regarding a loss of amino acids, but also, due to catabolic conditions, there is a deficit in the synthesis of several proteins [13,14,15,16]. AA (e.g., leucin) loss to dialysate results in a reduction in the net amino acid balance of about 12.3 umol/kg of body weight/1 h of treatment, i.e., the equivalent of a reduction in the protein synthesis of 5.8 g in a 4 h dialysis session in a 70 kg male [17]. This loss may be higher in high-flux dialyzers. During hemodialysis (HD), with the novel medium cut-off (MCO) dialyzers, there were median albumin losses of 2.9, 4.8, and 7.3 g per treatment session, depending on the type of MCO [18]. Moreover, until now, little attention has been paid to the impact of novel dialyzers on other biologically active proteins involved in the binding of hormones and drugs, because most studies on HD and HDF have focused on albumin loss. A recent study showed a significant decrease in alpha-1 acid glycoprotein (AGP) and vitamin-D-binding protein (VDBP) serum pre-dialysis levels after 3 months of MCO-HD. The dialysate albumin concentration was similar between MCO-HD and HF-HD [19,20].

It has been found that 30–50% of CKD patients have elevated levels of serum inflammatory biomarkers, e.g., C-reactive protein (CRP) and interleukin-6 (IL6). The etiology of inflammation in this case is varied and may be caused by underlying disease, comorbidity, oxidative stress, infections, obesity, and genetic or immunologic factors, or at the same time may be connected to hemodialysis-related factors, such as the dialysis membrane biocompatibility and dialysate type [21,22].

Peritoneal dialysis (depending on the type—intermittent peritoneal dialysis (IPD) or continuous ambulatory peritoneal dialysis (CAPD)) causes losses of approximately 9–19 g of protein and 5–15 g of amino acids per 10 h or 24 h treatment [23]. These losses have a huge impact on the transportation of many substances in the bloodstream and cells. Albumin transports a large amount of various small molecules, including lipids, bile pigments (bilirubin), vitamins, and some drugs. Inflammation and the blood purification methods used may additionally affect the increased demand for water-soluble vitamins [24,25,26].

### 1.5. Peritoneal Dialysis and Hemodialysis vs. Vitamins

Under conditions of increased levels of reactive oxygen species (ROS), ascorbic acid is oxidized to dehydroascorbic acid (DHA), which is transported by glucose transporters GLUT 1 and GLUT 3 to the interior of the mitochondrion [27]. Since the transportation of DHA via GLUT 1 and GLUT 3 competes with glucose, the uptake of dehydroascorbic acid into the mitochondrion is reduced [28]. Furthermore, the content of vitamin B1 in the plasma shows an inverse relationship with the level of glucose [29]

A mutation in the methylenetetrahydrofolate reductase (MTHFR) gene or a homozygous TT genotype additionally impairs the metabolism of vitamins B9, B12, and B6, as well as the conversion of homocysteine (Hcy) to methionine, resulting in increased Hcy levels associated with inflammation [30].

The loss of water-soluble vitamins and trace elements during hemodialysis has practically been a subject of research since the 1980s, when the first papers on this subject were written. The loss of water-soluble vitamins during dialysis depends on the characteristics of the dialyzer, the blood flow rate, the frequency of hemodialysis, and the composition of dialysis membranes [31]. The loss of these micronutrients is slightly different in patients dialyzed using classic hemodialysis and slightly different in patients on HDF dialysis.

What mechanisms are responsible for the loss of water-soluble vitamins? Is it diffusion or convection or plasma protein binding? This question is answered by the 2022 work of French authors. The authors of this original paper analyzed the results of patients undergoing post-dilution hemodiafiltration (HDF). The different levels of vitamins B1, B2, B6, B9, B12, and C; selenium; and zinc were tested. After hemodialysis, a significant decrease in the concentration of vitamins B1, B2, B6, B9, and C, as well as zinc, was found. There were no differences in the concentrations of B12 and selenium [32]. The proposed mechanism for the loss of these vitamins has been identified as diffusion and convection. Similar observations were made by Morena et al. based on vitamin C loss due to diffusion and convection during hemodiafiltration sessions [33], and Chazot made observations during conventional hemodialysis [34]. In both cases, these were prospective studies. Schwotzer investigated a group of patients undergoing HDF hemodialysis which received vitamin C and B complex supplementation. She described a decrease in vitamin C levels, as well as B1, B2, B6, and B6 levels, after the HDF hemodialysis session, but oral supplementation helped to maintain the level of vitamins in a proper range [35].

In conclusion, hemodialysis causes large losses of B1 and B2; therefore, this supplementation should be considered. Hemodialysis also causes a reduction in B6 and biotin levels in the blood. No specific syndromes associated with low pantothenic acid levels in the body have been reported in patients with CKD. Significant losses during each dialysis session are related to folic acid. Moreover, vitamin C levels decreased by 30–40%, but supplementation in high doses, especially when administered intravenously, increased oxalate levels in the blood. Plasma oxalate levels above 50 mcg/L can lead to tissue accumulation [36]. Folic acid supplements, especially when combined with vitamin B6 and vitamin B12 supplementation, can reduce elevated plasma homocysteine levels in patients with CKD, but do not improve clinical outcomes in patients with advanced CKD [37]. The guidelines of the European Society of Clinical Nutrition and Metabolism (ESPEN) recommend doses of water-soluble vitamin supplements at the recommended dietary allowance (RDA) level.

### 1.6. Diet Rich in Vitamins and Antioxidants in CKD

A diet based on plant products is rich in vitamins and antioxidants; therefore, it seems to be a good solution for patients with CKD. Nevertheless, the low content of wholesome protein may promote the loss of muscle mass, which will pose a risk to malnourished patients. Vegetables and fruits are an important source of bioactive ingredients and compounds, including antioxidants, vitamins (folic acid, vitamins C and E, carotenoids, and lycopene), flavonoids, indoles, and phenolic compounds. As well as this, lower intake levels of iron, pyridoxine, and cobalamin may be reflected in the severity of anemia in CKD patients [38]. Fat-soluble vitamins A and E are also important. They are potent dietary antioxidants that also have anti-inflammatory and antiapoptotic functions. The documented function of alpha tocopherol is an antioxidant function that scavenges peroxygen free radicals. The importance of this function is to maintain the integrity of long-chain polyunsaturated fatty acids in the membranes of cells and thus maintain their bioactivity [39]. In our previous studies, we showed fatty acid profile changes in CKD patients [40]. Additionally, oral vitamin A supplementation may be considered for the prevention of certain viral infections in CKD patients [41].

Research has shown that dietary fiber may lower serum urea levels due to increased nitrogen excretion from the gut. In turn, a diet low in fiber can increase the intestinal production of uremic molecules, such as p-cresol, indoxyl sulfate, and trimethylamine *n*-oxide (TMAO), secondary to the increased proteolytic activity of protein-fermenting bacteria. Dietary fiber significantly reduces blood urea nitrogen (BUN) levels. In the overall analysis, serum creatinine levels were significantly reduced. The authors found a significant effect of fiber consumption on the dose and response to the decrease in serum creatinine [42]. The consumption of large amounts of certain vegetables and fruits, due to their high potassium contents, may be associated with excessive levels in the plasma of patients. However, research from 2017 shows that the increase in serum potassium levels in patients who a lot of fruit and greens is insignificant [43]. Moreover, a vegetarian diet supplemented with ketoanalogs appears to be more beneficial in patients with more advanced CKD. Although dieting improves blood pressure control and proteinuria, as well as the main determinants of CKD progression, it also may contribute to a loss of muscle mass [3,44].

In studies that compare the use of three different types of diets, it was found that the Mediterranean dietary pattern (MDP), with a high consumption of vegetables (particularly beans, peas, and chickpeas), fish, olive oil, and eggs, and a low consumption of milk and red meat, was the most advantageous. A Western dietary pattern (WDP), with a high consumption of soft drinks, homemade fried potatoes, caffeinated beverages, and red and processed meats, and a low consumption of fruit and vegetable soups, along with diets low in animal protein (LAPp) but with a high intake of whole-grain bread, cookies and sweets, vegetable soups, and a low intake of white bread, rice, pasta, and potatoes, have been shown to be less beneficial to patients. Patients with an MDP showed lower sodium levels compared to those who had a WDP. Meanwhile, a higher lean tissue ratio and a lower body fat ratio were observed in patients with an MDP compared to those with an LAPp [45]. In summary, a diet rich in substances with antioxidant properties whose synergistic action supports metabolic processes and body cleansing is recommended for patients at all stages of CKD.

### 1.7. Anitioxidant Therapy

Oxidative stress is a major factor in the origin and worsening of chronic kidney disease. Many water-soluble vitamins have antioxidant properties; therefore, the level of other substances with these properties can be of great importance for the involvement and metabolism of vitamins from groups B and C [24]. It is therefore worth remembering a comprehensive system and the importance of supplying the body with antioxidants. Some authors believe that, in CKD patients, certain antioxidants can improve early kidney damage. Bolignano et al. point to the possible benefits of chronic antioxidant supplementations in diabetic kidney disease (DKD). Interventions included any antioxidant supplementation (e.g., vitamin A, vitamin E, vitamin C, methionine, ubiquinone, zinc, and selenium alone or in combination) [45]. Antioxidants remarkably decreased albuminuria levels compared to the controls, but did not have noticeable effect on renal function (GFR). The study was limited in the sense that only scarce information was available on hard endpoints (ESKDs), and there was high heterogeneity among studies with respect to the DKD severity, type, and duration of antioxidant therapy.

Another group of researchers focused on whether antioxidant therapy might reduce cardiovascular mortality and morbidity in people with CKD. Jun et al. assessed antioxidant therapy in hemodialysis patients, kidney transplant recipients, non-dialysis CKD patients (from mild to moderate stage); and patients requiring surgery [46]. Interventions included multiple antioxidant therapy, different doses of vitamin E, co-enzyme Q, acetylcysteine, bardoxolone methyl, and human recombinant superoxide dismutase. Antioxidant therapy showed no clear overall effect on cardiovascular mortality, all-cause mortality, cardiovascular disease, coronary heart disease, cerebrovascular disease, or peripheral vascular disease compared to the placebo. A limitation of this observation was the significant heterogeneity for cardiovascular disease when studies were analyzed by CKD stages. Antioxidant therapy for cardiovascular disease prevention in dialysis patients proved to be beneficial, but no such effect was observed in CKD patients. Antioxidant therapy was found to significantly reduce the development of end-stage of kidney disease, and it also lowered serum creatinine levels and improved GFR. Current evidence shows that treatment with antioxidants in patients with chronic kidney disease in the pre-dialysis period can prevent progression to ESKD, but this observation is based on a very small number of events.

Lactoferrin (LF) is an iron-binding glycoprotein with antioxidant, anti-inflammatory, and nitric oxide-dependent vasodilatory properties. While there is almost no evidence of clinical trials on LF in the treatment of kidney disease, preclinical research holds great promise given that LF has protective effects against acute kidney injury (AKI) and chronic kidney disease (CKD). Therefore, it is believed that drugs based on the LF nanocarrier can bring significant kidney protection [47].

In a large study, Khor et al. analyzed the efficacy of supplementation on inflammatory markers in hemodialysis patients [48]. Randomized controlled trials with different nutritional interventions were included in the review and were classified into antioxidants, vitamin D, fibers, polyphenol-rich foods, probiotics, and omega-3 fatty acids. Meta-analyses showed a significant reduction in CRP levels by omega-3 fatty acids and vitamin E. Evidence for other groups of nutrition supplements was inconclusive.

On the contrary, the most up-to-date meta-analysis, conducted by Lin et al., from 2020, showed that nutrition supplements in CKD patients did not appear to improve CKD prognosis [49]. The analysis of 17 randomized trials aimed to summarize and quantify evidence on the prevention effects of omega-3 polyunsaturated fatty acid (omega-3 PUFA), coenzyme Q10 (CoQ10), dietary fiber, vitamin D, and biotics (probiotics and prebiotics) on CKD progression. There seems to be very little evidence of the beneficial effects of these nutrition supplements in CKD patients on proteinuria, kidney function, and inflammations. The nutritional supplementation for CKD patients requires a discussion and consideration of the benefits over the adverse effects.

### 1.8. Uremic Microbiota and Intestinal Inflammation and Its Impact on Vitamin Absorption

Chronic kidney disease may be accompanied by the development of intestinal inflammation and epithelial barrier impairment, leading to the hastened systemic translocation of bacterial-derived uremic toxins [50]. Numerous studies have indicated that the progression of CKD to ESRD is strictly associated with the accumulation of toxic metabolites in blood. In ESRD patients, given the abundance of bacteria with urease, uricase, and enzymes that form indoxyl sulphate and p-cresyl sulphate, trimethylamine *n*-oxide is observed [51].

The numerous uremic solutes are generated in the process of protein fermentation by colonic microbiota. The elimination toxins reduced by impaired kidneys and the enhanced generation of toxins from the dysbiotic microbiome are the main reasons for the accumulation of uremic toxins. Additionally, phosphorus binders and ion exchange resins may slow intestinal transit, affecting the development of putrefactive microbiota in the colon [52]. Gut microbiota synthesizes vitamins K and B groups, the breakdown of indigestible plant polysaccharides, the activation of bioactive food components, the degradation of dietary oxalates, and the biotransformation of conjugated bile acids [50]. Dietary interventions comprising prebiotics, probiotics, and synbiotics could represent a promising strategy in the management of uremic toxins in CKD [53].

The following review article looks at the benefits and possible risks of supplementing water-soluble vitamins with the treatment of CKD. The authors conclude that despite the fact that antioxidant therapy does not reduce the risk of cardiovascular and all-cause death in CKD patients, it is possible that some may benefit from supplementation (particularly dialysis patients), which may also improve the status of water-soluble vitamins.

## 2. Material and Methods

**Figure 1:**
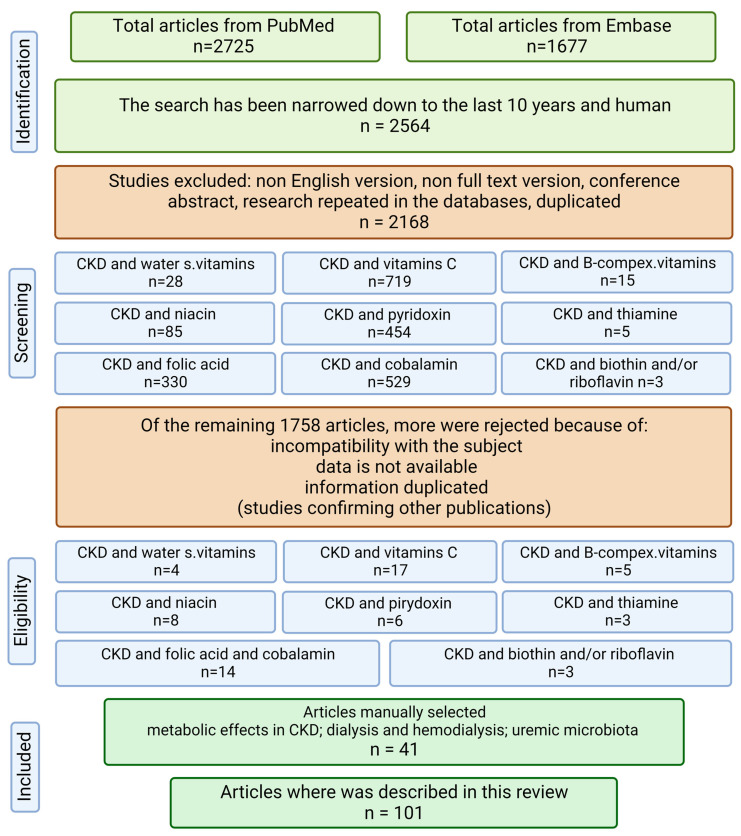
Flow chart of the review (created with BioRender.com https://app.biorender.com/, accessed on 2 February 2023).

The present review evaluates the above-mentioned topics based on the literature published 10 years prior to 20 July 2022. A systematic literature search was conducted in the PubMed and Embase databases. Only human studies were included in the review. The following keywords were searched: ((CKD) OR (chronic kidney disease) OR (dialysis) OR (hemodialysis) OR (renal failure) OR (kidney failure)) AND ((water-soluble vitamins) OR (vitamin C) OR (ascorbic acid) OR (B complex vitamins) OR (thiamine) OR (riboflavin) OR (niacin) OR (vitamin B6) OR (pyridoxine) OR (folate) OR folic acid OR (vitamin B12) OR (biotin) OR (pantothenic acid) OR (cobalamin)). Studies that were not in the English language, letters to the editor, and abstracts of conferences were excluded, as shown in the flow chart (Figure 1).

## 3. Discussion—Supplementation with Water-Soluble Vitamins

CKD can develop at different rates and severity levels in different patients, and this phenomenon can be caused by genetic variants of inflammation, including variant rs883484, located upstream of the prostaglandin endoperoxide synthase 1 (PTGS1) gene [53]. Some groups of patients, e.g., minor homozygotes of rs883484 who take in niacin, α-tocopherol, and vitamin C, may benefit from this intervention as a lower CKD tendency in this group of patients was found, which was confirmed in a population-based cross-sectional study of 684 Japanese participants.

Interesting conclusions were made by Rafeq et al., who observed that therapy with high doses of B vitamins in CKD patients, despite the reduction in plasma homocysteine, was associated with the exacerbation of left ventricular diastolic dysfunction in patients with advanced CKD (GFR < 30 mL) or end-stage renal disease (ESRD) [54]. The origin of this phenomenon is unclear. Preclinical animal studies show that altering the methionine–homocysteine cycle has a direct effect on the heart muscle. This leads to increased fibrosis and collagen deposition, and ultimately to myocardial stiffening and pump failure [55].

**Figure 2:**
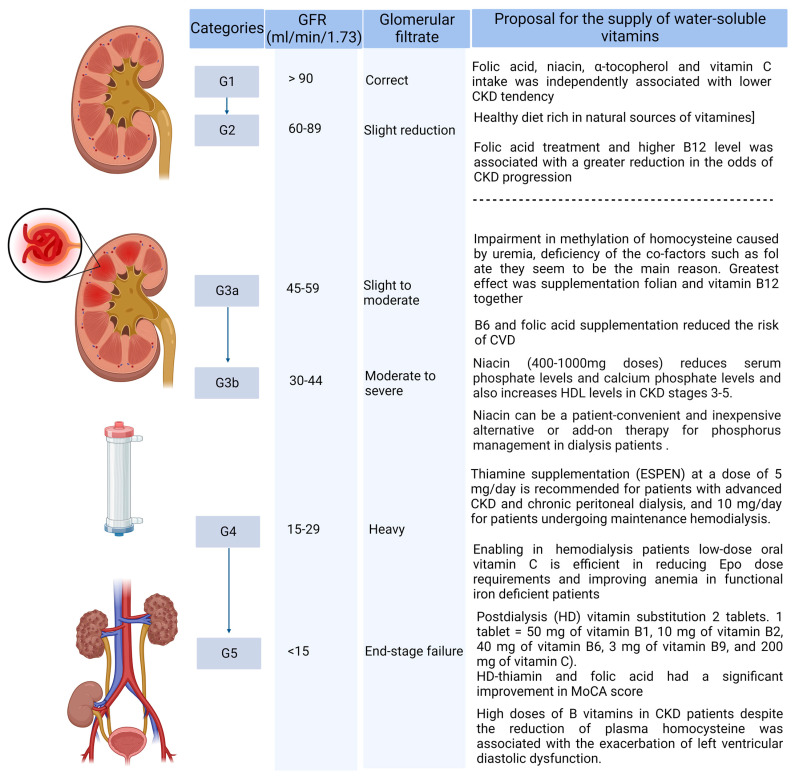
Supply of water-soluble vitamins, taking into account the CKD stage. (Created with BioRender.com https://app.biorender.com/, accessed on 2 February 2023.)

The influence of hemodialysis (HD) on the level of hydrosoluble vitamins B and C was also investigated. It was found that the deficiency of hydrosoluble vitamins B and/or C is rare in patients on HD and only occurs in patients who do not take a post-dialysis vitamin substitution [56]. Therefore, taking two tablets after the HD procedure (one tablet = 50 mg of vitamin B1, 10 mg of vitamin B2, 40 mg of vitamin B6, 3 mg of vitamin B9, and 200 mg of vitamin C) in the 3-month study prevented plasma deficits in patients [57]. A summary of the supplementation of water-soluble vitamins in CKD patients is presented below (Figure 2).

### 3.1. Vitamin C Supplementation

In the early stages of CKD, where it is advisable to eat a low-protein diet high in vegetables and fruits, the need for vitamin C and other antioxidants is fully covered. The content of vitamin C in a properly composed diet, depending on the products used, is 100–400 mg/day. In patients with advanced CKD undergoing dialysis therapy and whose diet often deviates from the correct one, a question arises about using supplementation.

Though fears have been dispelled that alternate-day hemodialysis can decrease the concentrations of water-soluble vitamins and adversely affect patients’ well-being, the use of vitamin C supplementation in patients with advanced kidney disease seems necessary for other reasons [57].

Chronic inflammation is one of the leading causes of cardiovascular disease in hemodialysis patients. Vitamin C, in turn, is the main antioxidant that can effectively affect inflammation [57].

Vitamin C emerged as a potential therapy to improve the treatment of anemia by increasing iron mobilization. Vitamin C may be used as an adjuvant therapy in functional iron deficiency. It has been shown that vitamin C therapy may be beneficial by lowering serum hepcidin and hs-CRP levels in patients on hemodialysis with so-called functional iron deficiency anemia [58]. Conner et al. observed higher plasma concentrations of F2-isoprostanes, IL-1, IL-10, and TNF-α after vitamin C infusion [59]. However, the administration of high doses of vitamin C (50 mg/kg, four times a day) intravenously carries a high risk of oxalate nephropathy [58]. Long-term safety studies of such therapy (intravenous iron plus vitamin C) are required [60].

In smaller doses (300 mg 3 times/week), ascorbic acid increases the amount of iron available for erythropoiesis and improves the correction of anemia [3,61].

Moreover, contrast-induced nephropathy (CIN) has been defined as a significant impairment of kidney function within 2 to 3 days after the administration of the contrast agent, and the use of ascorbic acid for the prophylaxis of CIN is listed in international guidelines as a potential benefit agent [62,63]. Supplementation was also carried out in a group of children with an average age of 12 years at a dose of 250 mg, observing an increased level of vitamin C in the plasma without increasing serum oxalate [64]. Oral vitamin C in doses of 250 mg/day effectively reduced anemia and the need for a dose of Epo in patients with functional iron deficiency, without the need for additional iron administration [65,66]. An extensive meta-analysis from 2019 showed that ascorbic acid (AA) supplements were remarkably correlated with a higher risk for kidney stone incidence in men but not in women [67].

It was also shown that vitamin C was an effective and safe supplementation for the treatment of restless leg syndrome (RLS) in hemodialytic patients [68,69]. End-stage kidney disease causes and impaired immunity, and this is why ESKD patients are in the high-risk group for coronavirus disease 2019 (COVID-19). Therefore, the inclusion of vitamin C infusions, despite the imminent consequences, can be used as supportive therapy [70,71]. Studies on supplementation with ascorbic acid are presented in the table below (Table 1).

### 3.2. Supplementation with Folate and Cobalamin

In both CKD and ESRD patients, several metabolic changes, including metabolic acidosis, systemic inflammation, and hormonal disturbances, along with comorbidities and multi-drug therapy, can lead to malnutrition, followed by folate and vitamin B12 deficiency [1]. For patients in the early stages of CKD for whom there is no indication to limit their intake of a diet rich in vegetables and fruits, folic acid may be in the form of a healthy diet rich in natural sources of folic acid [1].

Hyperhomocysteinemia (HHcy) is a risk factor for cardiovascular disease, particularly in patients with end-stage renal disease (ESRD). HHcy is reported in 80–100% of HD patients [72]. This is mainly due to impairment in the methylation of homocysteine caused by uremia, but the deficiency of co-factors, such as folate, seems to be the main reason [73]. The results of this study show that, overall, the greatest effect of vitamin B12 supplementation on lowering homocysteine levels in ESRD patients was when combined with folate supplementation [74]. Folates exert a direct antioxidant effect by affecting cellular oxidative metabolism, restoring the proper function of the endothelium. It has been shown that injection treatment with folates may have an advantage over oral supplementation [73]. Unfortunately, it was also shown that supplementation with folic acid may not have an effect on mortality in patients with CKD, but the certainty of the evidence is low. In addition, supplementation with folates does not reduce the risk of cardiovascular events or stroke in patients with CKD [75].

It was found that among hypertensive patients, folic acid therapy reduces the risk of mortality associated with heavy proteinuria [76]. Liu et al. reported on the American population (in a thirty-year observation) that higher folate intake in young adulthood was longitudinally associated with a lower incidence of CKD later in life [77]. Patients with lower or excess folate status both face greater mortality risks. Serum folate concentrations may be non-linear in the CKD population, indicating a reference range of 14.7–19.1 ng/mL with the best survival outcome [78]. Other studies on folic acid supplementation are presented in the table below [79,80,81,82] (Table 2). The efficacy of folic acid therapy in the progression of CKD in the Chinese population was demonstrated [83]. Meanwhile, vitamin B12 deficiency should be addressed in ESRD patients receiving HD [84] (Table 2).

### 3.3. Pyridoxine Supplementation

Vitamin B6 deficiency is common in hemodialysis patients and may contribute to abnormal bone metabolism and anemia. Moreover, vitamin B6 deficiency may engender immune dysfunction, inflammation, increased oxalate generation, and polyneuropathy. Dietary intake is reduced as the disease progresses. Other researchers have reported low vitamin B6 intake in patients with advanced CKD and chronic dialysis [85]

The overall incidence of vitamin B6 deficiency was 40%. Vitamin B6 (60 mg of pyridoxal 5′-phosphate hydrate), given intravenously three times a week, worsens the response to ESA (resistance to erythropoiesis stimulants) and may reduce the bone response to parathyroid hormones in hemodialysis patients. One must be careful with high doses of supplementation [86]. Schwotzer, N., et al. showed that the loss during hemodialysis was 27% [57]. As for the benefits of vitamin B6 supplementation in CKD, they have been studied in the context of associations with other B vitamins, and the available studies report various results that are often contradictory [87]. Treatment with folic acid, vitamin B6, and vitamin B12 does not reduce cardiovascular risk or mortality in patients with CKD [88].

### 3.4. Niacin Supplementation

Niacin has a well-documented beneficial effect in the treatment of hyperlipidemia, as it was the first to be registered as a lipid-lowering agent [89]. It most effectively lowers serum triglyceride as well as low-density lipoprotein cholesterol (LDL-C) levels, and effectively raises high-density lipoprotein cholesterol (HDL-C) levels.

Hyperphosphatemia is common in patients with end-stage renal disease. Niacinamide and niacin have been shown to cause clinically significant reductions in serum phosphate levels in dialysis patients [90]. As is known, hyperphosphatemia is an important risk factor not only for secondary hyperparathyroidism, but also in CVD. Dietary phosphorus restriction and phosphorus removal by dialysis have been shown to be insufficient to control serum phosphate levels [91,92].

Consequently, it becomes necessary to use phosphate binders to reduce absorption from the intestine [93]. Current therapies have their burdens, such as hypercalcemia (e.g., calcium carbonate) or aluminum toxicity (aluminum hydroxide), sevelamer, and lanthanum carbonate.

There is a need for phosphate-lowering agents that overcome some of the drawbacks of current therapies. Niacin has a potential advantage over the current phosphate binders, as it does not need to be administered with a meal [94].

Currently, niacin is rarely used in clinical settings, because statins are indicated in cardiological recommendations for the treatment of lipid disorders [95]. The use of niacin has been further limited after a failed trial which led to the suspension of the niacin-laropiprant (redness blocker) combination product from the world market [94].

However, it seems that patients with chronic kidney disease may benefit from the use of niacin thanks to its additive antilipemic effect. It seems that niacin may be a convenient and inexpensive alternative or additional therapy in lowering phosphorus in dialysis patients [96].

The most common adverse events were cutaneous flush, nausea, and diarrhea. Approximately 25% of these patients had early and mild adverse events [96]. The studies conducted so far on niacin supplementation in CKD are presented in the table below (Table 3).

### 3.5. Supplementation with Thiamine

Vitamin B1 (thiamine) is responsible for supporting metabolic processes and the nervous system. It is naturally found primarily in grain products and legumes. The rate at which this vitamin is lost from the patient’s reserves begins long before the onset of clinical symptoms. CKD results in a marked down-regulation in the expression of thiamin transporters in the intestine, heart, liver, and brain [97]. Therefore, decreased intestinal absorption and cellular homeostasis impairment levels were expected as a result of this deficit.

Vitamin B1 losses during hemodialysis are quite high, as high as 49% in one session [52]. The first atypical symptom of thiamine deficiency in these patients is consciousness disturbance. Patients undergoing hemodialysis with cognitive impairment treated with thiamine and folic acid have shown significant improvements in the MoCA score (assessment of the improvement in cognitive functions). A daily thiamine supplementation that complies with the RDA (1.5 mg or 0.8 mg) for healthy subjects is indicated in dialysis and is sufficient to keep thiamin status within the normal range [98]. The serum thiamine concentration drops by 27% with one dialysis session. According to ESPEN and CARI recommendations, thiamine supplementation at a dose of 5 mg/day is recommended for patients with advanced CKD and chronic peritoneal dialysis, and 10 mg/day for patients undergoing maintenance hemodialysis [52].

There are no data on the recommended doses of vitamin B1 supplementation in hemodialysis patients. Due to the risk of developing severe encephalopathy during dialysis, patients may benefit from low-dose prophylaxis during conservative treatment [77].

### 3.6. Supplementation with Biotin

Biotin occurs in free or protein-bound forms. Its deficiency is exacerbated by the lack of appetite, which is already lowered in people with end-stage kidney disease. Biotin naturally occurs primarily in meat, fish, and eggs, i.e., protein products that require a high consumption restriction. Biotin losses in one dialysis are only about 12%. Vitamin levels remain within the reference range after lowering the usual dose of supplementation. Biotin deficiency is rare in patients with end-stage kidney disease [56]. Vitamin B7 supplementation, however, is one way of reducing the incidence of muscle cramps in hemodialysis patients, irrespective of the plasma biotin concentration. This concentration may even be overestimated due to the presence of biotin metabolites that do not function as a vitamin [99]. In hemodialysis patients, biotin supplementation may result in a sharp increase in the concentration of 25-hydroxyvitamin D and a decrease in parathyroid hormone levels; therefore, these parameters should be monitored when using possible supplementation [100]. There are no unequivocal literature data which indicate the need for biotin supplementation in patients with CKD.

### 3.7. Supplementation with Riboflavin

The studies involving riboflavin supplementation in CKD have so far received little attention. However, it seems that due to the fact that dairy products are the main source of this vitamin, its deficiency may be a significant threat. As a flavin coenzyme (FAD and FMN), riboflavin is used to metabolize proteins, carbohydrates, and fats in order to generate energy, but also as an antioxidant to maintain the normal functions of the immune system. Currently, over 100 flavin enzymes, e.g., glutathione reductase, may affect the lifespan of erythrocytes, which, in the case of malnourished and/or anemic patients, may be essential in supporting therapy [101].

## 4. Limitations and Future Directions

The supplementation of water-soluble vitamins in CKD patients is an important element of therapy, preventing the progression of the disease, especially starting from the moderate CKD phase. In order to conduct research in this area, the intake of vitamins along with the diet and the intake of other substances with antioxidant properties should be taken into account. A consideration of the patient’s protein nutritional status and albumin level may be required.

The largest meta-analyses concerned the Asian population; therefore, additional population studies in other cohorts may be required. Many issues have not yet been resolved. The literature shows that the relationship between CKD, heart disease, and vitamin deficiency is mainly related to vitamin K, vitamin D, and magnesium deficiency. There is no information on the content of water-soluble vitamins, which may be an area for further research. Research has not been conducted in recent times to supplement biotin and riboflavin in CKD, which may represent a new direction for the future.

## 5. Conclusions

In carrying out studies on vitamin intake, it was found that higher intakes of folate, niacin, tocopherol, and vitamin C reduced the risk of CKD. Therefore, a diet rich in plant products that are rich in natural antioxidants should be used for prevention purposes.

When analyzing the need for supplementation with water-soluble vitamins, taking into account the CKD stages, it was found that in the initial period of failure (G2-G3a) it does not require intervention, but later, especially in the case of inadequate nutrition, the inclusion of supplementation with folates and cobalamin may bring various benefits. Such supplementation seems to be a necessity in patients with uremia. Conversely, the inclusion of additional B6 supplementation to reduce CV risk may be considered.

In stage 3b and beyond (stages 4–5), the inclusion of niacin in a dose of 400–1000 mg depending on the patient’s tolerance is required in order to lower the phosphate level. The inclusion of supplementation with thiamine and other water-soluble vitamins, especially in peritoneal dialysis and hemodialysis patients, is necessary in order to reduce dialysis losses. However, it should be considered that doses of B vitamins several times higher than the RDA of consumption may be the cause of the exacerbation of left ventricular diastolic dysfunction in CKD patients.

## Figures and Tables

**Table 1:** Studies on ascorbic acid supplementation in CKD.

Author, Year	Patient	Supplementation	Effect
Conner, 2012 [59] (prospective study)	N = 13	Hemodialysis (HD) patients: 300 mg of vitamin C	Observed higher plasma concentrations of F2-isoprostanes, IL-1, IL-10, and TNF-α post-infusion
Sedighi, 2013 [61] (RCT study)	N = 40	300 mg of vitamin C post-dialysis, twice a week for 5 consecutive weeks, with a follow-up period of 12 weeks	Equal efficacy for intravenous iron and intravenous vitamin C for treatment of anemic HD patients with serum ferritin ≥500 mg/mL and TSAT ≤ 25%
Biniaz, 2014 [57] (retrospective studies)	N = 151	Intervention: 250 mg of vitamin C after hemodialysis session 3 times a week for 8 weeks	After 2 months, median CRP reduced significantly in the vitamin C group to 10.7 (*p* = 0.04) vs. 22.6, and 30.6 mg/L in control groups
Garneata, 2015 [3] (RCT study)	N = 69	Hemodialysis patients: ascorbic acid (300 mg 3 times/week) for 12 months intravenously	Ascorbic acid increased the amount of iron available for erythropoiesis and improved the correction of anemia
Rafie, 2016 [68] (RCT study)	N = 45	HD patients: 250 mg of vitamin C daily for 8 weeks	Ascorbic acid was shown to be an effective and safe supplementation for the treatment of RLS in hemodialytic patients The treatment was not accompanied with serious adverse effects in short-term follow-up
Sultana, 2016 [65] (prospective study)	N = 15	HD patients: 250 mg oral vitamin C daily for 3 months	Low-dose oral ascorbic acid was efficient in reducing erythropoietin dose requirements and improving anemia in functional iron deficient patients, without requiring additional iron administration
Ali, 2021 [58] (RCT study)	N = 31	Treatment of erythropoietin-stimulating agents (ESAs), together with the oral supplementation of 500 mg of ascorbic acid every other day for 3 months in addition to iron therapy	Study group: reduction in both hepcidin and hs-CRP levels; reduction in serum iron and ferritin levels (*p* < 0.05); correlation between serum hepcidin and hs-CRP (R = 0.46, *p* <0.01)

RCT (randomized control trial) study.

**Table 2:** Studies on folic acid and cobalamin supplementation in CKD.

Author, Year	Patient	Supplementation	Effect
Qin, 2013 [79] (meta-analysis, multicenter studies)	N = 8234	Folic acid supplementation; B12 and folic acid supplementation; B6 and folic acid supplementation	Folic acid therapy reduced the risk of CVD in patients with kidney disease by 10%. Folic acid alone vs. folic acid with vitamin B6 and B12 did not significantly affect the effect of folic acid therapy.
Rafeq, 2013 [54] (RCT study)	N = 220	Post hoc analysis HOST *	High-dose B vitamin therapy may be harmful in patients with CKD.
Xu, 2016 [83] (RCT, multicenter studies)	N = 7545	0.8 mg of folic acid; follow-up for an average of 4.5 years	Enalapril–folic acid therapy significantly slowed down the progression of CKD in patients with hypertension (compared to enalapril alone).
Achour, 2016 [72] (RCT study)	N = 132	Cyanocobalamin (ampoule 1 mL/intramuscular injection of siphat) and folicum (folic acid, 5 mg/tablet) for 6 months	Supplementation with B vitamins (B9 and B12) correlated to the MTHFR genotypes was shown to significantly lower tHcy in HD patients regardless of MTHFR 677 genotype. Flushed out of the body after 2 months.
Li, 2017 [76] (RCT, multicenter Studies)	N = 20,702	10 mg enalapril or 10 mg enalapril and 8 mg folic acid (median treatment duration of 4.5 years)	Folic acid supplementation significantly reduced the risk of all-cause mortality in patients with heavy proteinuria, but not in those with absent or mild proteinuria.
Li, 2020 [80] (RCT, multicenter studies)	N = 1374	10 mg of enalapril or 10 mg of enalapril and 0.8 mg of cobalamin (median treatment duration of 4.4 years)	Folic acid treatment was associated with a greater reduction in the odds of CKD progression among patients with mild-to-moderate CKD and higher B12 levels.
Lu, 2021 [81] (RCT study)	N= 25	Thiamin 90 mg/day combined with folic acid 30 mg/day for 96 weeks	Hemodialysis patients with cognitive impairment treated with thiamin and folic acid had a significant improvement on the MoCA score.
Lydia, 2021 [82] (cross-sectional study)	N = 80	B12 and folic acid supplementation; no data (medical history)	There is a significant negative correlation between vitamin B12 and folic acid with homocysteine levels, especially in the high-risk cardiovascular group.
Yan, 2021 [78] (retrospective multicenter study)	N = 2142	Levels of folic acid analyzed in the plasma	Reference range of folic acid in plasma was 14.7–19.1 ng/mL, with the best survival outcome in patients with CKD.
Nahas, 2022 [84] (prospective study)	N = 110	B12 supplementation (1 mg once weekly for the next 4 weeks and 1 mg at the end of the second month)	Vitamin B12 deficiency should be addressed in ESRD patients receiving HD, while vitamin B12 supplementation may provide promising positive outcomes in the management of renal anemia among this population.

* HOST— homocysteinemia in kidney and end-stage renal disease; RCT (randomized control trial) study.

**Table 3:** Studies on niacin supplementation in CKD.

Author, Year	Patient	Supplementation	Effect
Takahashi, 2004 [89] (prospective study)	N = 65	500 mg of niacinamide for 12 weeks	Serum phosphorus decreased from 6.9 ± 1.5 to 5.4 ± 1.3 mg/dL. HDL increased from 47.4 +/− 14.9 to 67.2 +/− 22.3 mg/dL. LDL concentration decreased from 78.9 +/− 18.8 to 70.1 +/− 25.3 mg/dL.
Sampathkumar, 2006 [91] (prospective study)	N = 34	375 mg of nicotinic acid for 8 weeks	Serum phosphorus decreased from 7.7 ± 1.5 to 5.6 ±1.1 mg/dL. Calcium increased from 8.1 ± 1.0 to 8.5 ± 1.0 mg/dL. Serum alkaline phosphatase decreased from 107 ± 66 IU/L to 82 ± 46 IU/L.
Muller, 2007 [72] (prospective study)	N = 17	375 mg/dL to 2000 mg/dL of nicotinic acid (Niaspan), showing a systematic change every 2 weeks for 12 weeks	Serum phosphorus decreased from 7.2 +/− 0.5 to 5,9 +/− 0.6 mg/dl. HDL increased from 40 +/− 3.2 to 59 +/− 5.5 mg/dL. No effect on serum calcium levels.
Restrepo Valencia, 2008 [93] (observational study)	N = 9	500 mg of nicotinic acid for 3 months and 1000 mg for 5 months	Serum phosphorus decreased from 6.46 ± 0.53 to 3.94 ± 0.76 mg/dL. HDL increased, but there were no changes in LDL, PTH, hemoglobin, platelet count, AST, ALT and bilirubin, clotting tests (TTP and TP), uric acid, glycemia, albumin, creatinine, BUN, ferritin, folic acid, or vitamin B12.
Maccubbin, 2010 [94] (RCT study)	N = 1547	1 g/d of inhibitor laropiprant for 4 weeks and dose advanced to 2 g/d for 20 weeks combined with the selective prostaglandin D2 receptor subtype 1	A sustained 0.13 mmol/L (0.4 mg/dL) reduction in serum phosphorus concentrations, approximately 10% from baseline, which was unaffected by estimated GFR levels ranging from 30 to > or =90 mL/min per 1.73 m(2).
Edalat-Nejad, 2012 [90] (RCT study)	N = 37	400 mg of niacin for 2 weeks, 600 mg for 2 weeks, 800 mg for 2 weeks, and 1000 mg for 2 weeks (8 weeks in total)	Serum phosphorus decreased from 6.66 ± 1.40 to 5.96 ± 0.87 mg/dL. HDL cholesterol increased from 35.3 ± 7.26 to 40.6 ± 10.1 mg/dL.
Edema, 2021 [96] (RCT study)	N = 89	500 mg of niacin for 4 weeks and 1000 mg for 4 weeks	Niacin reduced serum phosphate levels and calcium phosphate levels and also increased HDL levels in CKD stages 3–5 patients.

RCT (randomized control trial) study.

## Data Availability

Not applicable.
